# Education of the People about the Teeth

**Published:** 1897-05

**Authors:** S. M. Fowler

**Affiliations:** Muskegon, Mich.


### Education of the People about the Teeth.

BY S. M FOWLER, D.D.S., MUSKEGON, MICH.

The subject I have selected to present to this meeting is one I consider of great importance. That there is need for more extended education among the masses in matters pertaining to the teeth, I think will be conceded by every one who has an opportunity to realize how little people in general know of the development and care of their teeth. I think all intelligent dentists will agree with me that the more intelligent the patients in regard to their teeth, the more satisfactory patients they make and the more your honest conscientious efforts in their behalf are appreciated. I have no disposition to undervalue the satisfaction that comes to the dentist by having his patient, upon the completion of his work, make out a check in your favor for the slight trouble he has put you to, but how much more satisfied you feel if that same patient knows and appreciates that you have given him good, honest service, and all you have done has been for his good.

But when we see people who are intelligent on other matters, people who are very solicitous about their general health, who insist on the very best hygienic surroundings, who are very careful of their diet and who must retire and rise at a certain hour, I say, when we see these people totally ignore the fact that in failing to take care of their teeth, they are violating one of Nature's strictest laws. We are astonished and we feel that something should be done, some step taken to educate the masses in this direction, and of course, we cannot expect others to take hold of this matter so long as those who have made this a special study and life's work keep silent.

We are surrounded by many peeple who would gladly come to us once a year and have their teeth kept in the best possible condition if they were only sufficiently impressed with the importance of such a procedure. We will admit that one of the best places to educate is in our own office by taking the young child and pointing out to the mother which of the teeth are permanent and which are temporary, and how long the temporary ones should be retained. But when you take the little one into your chair, and point out to the mother the sixth-year molar and tell her that is a permanent tooth and should be preserved, how many, many times will you hear the exclamation, "Oh! Doctor, you must certainly be mistaken; why, that tooth has just come and I know she has never had a tooth extracted there," and will call upon the little one to corroborate her statement, and that settles it, for surely had the little one ever had a tooth extracted she would remember it. But educating in your office has its drawbacks, for I care not how high your standing in your community may be, or what your reputation for trúth and veracity may be, there will still be a lurking suspicion in the minds of some that there's "method in your madness," when you recommend this tooth filled with gold, that tooth filled with cement, or that molar or bicuspid crowned.

A great many dentists send out to their patients small booklets containing a good deal of valuable information and would be great educators if they were only read. But almost invariably they have printed on the outside covers, "Compliments of Dr.———." These are at once classed by those in whose hands they chance to fall as an advertisment and boom for Dr.———, and while I have no criticism to offer on the idea, yet, I dare say, they are read by very few people.

But we all have at hand a practical, effective and strictly legitimate means of extending dental education to the masses, and that, too, at an age when such information is most needed, where it will do the most good, and in a manner that will be most apt to be heeded.

In early childhood this education should begin, and every child can be reached very nicely and very easily through our public schools.

In our State we have two organizations of the teachers—The Teachers' Association and The Teachers' Institute; the one meets once a year for a three days' session and the other meets twice a year for one day. At these meetings all matters looking toward the advancement of our schools are discussed, papers are read on the various branches taught, and frequently our business and professional men are invited to read a paper, or lecture on matters pertaining to their profession. At one of these meetings I was invited to read a paper on a subject pertaining to my profession; I prepared a paper on the development and care of the teeth, and the results were most gratifying. This was something new to them, and they declared had been greatly neglected in the schools. But it was one in which they manifested great interest, and the next Winter I was invited to prepare a second paper, which, I take it, was the very best evidence that the subject was one they desired to know more about.

Before I began, I said to them that after reading my paper, if it suggested any queries to them, I would be glad to answer any questions they might wish to ask. And right here let me tell you, as a matter of information, that before giving 200 teachers license to ask you any questions your paper may suggest to them, it will save you a deal of embarrassement, if you study well the subject you present for I assure you that you will have questions asked that have never occurred to you, and you will be expected to answer them promptly.

In this way your ideas will go directly to the youth of your county, for when one of those teachers comes to the subject of the teeth in her Physiology class, she will remember a great many of your suggestions, and will be a great deal better prepared to teach the subject than she was before she listened to you.

By pursuing this course, I feel that it will be but a short time before our patients will be much more intelligent on this subject; they will come to us with better ideas of what can be done for them, a better conception of what ought to be done, and they will certainly appreciate your efforts.

This is a duty we owe to ourselves, we owe it to the profession we represent and we owe it to the community in which we live.

If you will try it you will not only get the satisfaction that comes to you by having intelligent patients, but you will have the greater satisfaction that comes in the knowledge of having helped to confer a most beneficent boon on mankind in general.

